# Zinc and Regulation of Inflammatory Cytokines: Implications for Cardiometabolic Disease

**DOI:** 10.3390/nu4070676

**Published:** 2012-07-04

**Authors:** Meika Foster, Samir Samman

**Affiliations:** Discipline of Nutrition and Metabolism, School of Molecular Bioscience, University of Sydney, Sydney, NSW 2006, Australia; Email: meika.foster@sydney.edu.au

**Keywords:** zinc, inflammation, cytokines, atherosclerosis, diabetes mellitus

## Abstract

In atherosclerosis and diabetes mellitus, the concomitant presence of low-grade systemic inflammation and mild zinc deficiency highlights a role for zinc nutrition in the management of chronic disease. This review aims to evaluate the literature that reports on the interactions of zinc and cytokines. In humans, inflammatory cytokines have been shown both to up- and down-regulate the expression of specific cellular zinc transporters in response to an increased demand for zinc in inflammatory conditions. The acute phase response includes a rapid decline in the plasma zinc concentration as a result of the redistribution of zinc into cellular compartments. Zinc deficiency influences the generation of cytokines, including IL-1β, IL-2, IL-6, and TNF-α, and in response to zinc supplementation plasma cytokines exhibit a dose-dependent response. The mechanism of action may reflect the ability of zinc to either induce or inhibit the activation of NF-κB. Confounders in understanding the zinc-cytokine relationship on the basis of *in vitro* experimentation include methodological issues such as the cell type and the means of activating cells in culture. Impaired zinc homeostasis and chronic inflammation feature prominently in a number of cardiometabolic diseases. Given the high prevalence of zinc deficiency and chronic disease globally, the interplay of zinc and inflammation warrants further examination.

## 1. Introduction

Zinc was established as an essential trace element in 1961 following the discovery of zinc deficiency in humans [1]. It is one of the most abundant elements within cells and is necessary for a broad range of physiological processes. Zinc is an integral component of proteins involved in cell structures and the stabilisation of cell membranes. It functions to maintain the structural integrity of as many as 3000 transcription factors in the human genome and is essential for the biological activity of more than 300 zinc metalloenzymes [2]. In addition to its numerous structural and catalytic functions, zinc is involved in the regulation of an extensive variety of genes, impacting such diverse processes as protein-protein interactions, nucleic acid metabolism, cell replication, apoptosis, and signal transduction [3]. The small proportion of readily exchangeable or “free” zinc within cells [4] recently has been ascribed neurotransmitter functions [5], highlighting the diverse roles of zinc in biology.

Zinc deficiency is reported to contribute significantly to the global burden of disease [6]. Although severe zinc deficiency is relatively rare in developed countries, based on population estimates of dietary zinc intake less acute deficiency states are believed to be highly prevalent [7]. In addition to inadequate dietary zinc intake, deficiency may result from impaired absorption or resorption or increased excretion of zinc; conditions such as chronic diarrhea, extensive burns, or traumatic and surgical wounds increase endogenous zinc losses. An initial consequence of zinc deficiency is an impairment of immunological functions [8]. The wide involvement of zinc in the immune system [9] includes an ability to influence the production and signalling of numerous inflammatory cytokines in a variety of cell types [10,11] (Table 1). 

**Table 1:** Selected cytokines: cell sources and examples of their principal functions in inflammation.

Cytokine	Primary Cell Sources	Key Functions in Inflammation
IL-1	Macrophages Endothelial cells	Synthesis of acute phase proteins by hepatocytes; Local and systemic inflammatory effects
IL-2	Activated T cells Th1 cells	Proliferation of T cells, B cells; Proliferation and activation of NK cells
IL-6	Macrophages Th2 cells Endothelial cells Adipocytes Myocytes Osteoblasts	Synthesis of acute phase proteins by hepatocytes; Proliferation of B cells; Down-regulation of IL-1 and TNF production; Activation of immune cells, osteoclasts, endothelial cells; Hypothalamic Pituitary Axis—fever & hormone release
IL-10	MacrophagesTh2 cells	Resolution of inflammation; Inhibition of Th1 inflammatory cytokine synthesis; Inhibition of activated macrophages and dendritic cells
IL-12	Macrophages Dendritic cells	Promotion of Th1 differentiation; Stimulation of IFN-γ production by T cells, NK cells
TNF-α	Macrophages T cells NK cells Lymphoid cells Endothelial cells Adipocytes Cardiac myocytes Fibroblasts Neuronal cells	Synthesis of acute phase proteins by hepatocytes; Recruitment and activation of neutrophils and monocytes at sites of infection; Stimulation of CRP release from liver; Activation of NF-κB pathway; Induction of insulin resistance
TGF-β	Macrophages T cells	Resolution of inflammation; Limit production of IL-2, IFN-γ, and TNF; Inhibition of proliferation/activation of B cells, T cells, macrophages.
IFN-γ	Th1 cells NK cells	Activation of macrophages; Suppression of Th2 cell activity; Promotion of leukocyte migration

Abbreviations: IFN, interferon; IL, interleukin; NK, natural killer; NF-κB, nuclear factor-kappaB; Th, T helper; TGF, transforming growth factor; TNF, tumour necrosis factor [10,11].

Inflammation (INF) is an integral part of the innate immune system’s response to trauma or infection. The acute inflammatory response is initiated upon detection of inducers, such as microbial infections, oxygen radicals, and tissue damage, by sensors such as Toll-like receptors (TLR) and other pattern recognition receptors. The purpose of INF is to protect the host from the spread of infection or tissue damage, whereupon the inflammatory response is resolved and homeostasis is restored. In typical cases, the inflammatory response is localised to the site where the inflammatory inducer is present; however an increasing number of inflammatory conditions have been described where the initiating trigger is not well defined and INF appears to be chronic [12,13]. The redistribution of zinc in such conditions affects zinc homeostasis [14]. The aim of this review is to evaluate the literature on the interactions of zinc and cytokines in cardiometabolic disease. The presence of low-grade systemic INF in conjunction with perturbed zinc homeostasis in chronic disorders, such as atherosclerosis and diabetes mellitus (DM), highlights a role for zinc nutrition in the management of cardiometabolic symptoms [15].

## 2. Zinc Homeostasis and Inflammation

In humans, homeostatic mechanisms maintain plasma zinc within a concentration range of approximately 10 to 18 μmol/L. Cells are dependent on plasma to supply them with a constant supply of zinc to sustain normal function. In zinc deficiency, immune cells may be the first to respond to a change in zinc status even before plasma zinc concentrations fall below the normal range [16]. Cellular zinc concentrations are maintained by two classes of zinc transporters: the ZnT (SLC30) and Zip (SLC39) zinc transporter families. ZnT transporters promote cellular zinc efflux or its sequestration into intracellular organelles; conversely, Zip transporters facilitate extracellular or organellar zinc influx into the cytoplasm. Metallothionein (MT) also is believed to play a central role in the maintenance of zinc homeostasis by trafficking zinc through the cell and releasing it to zinc-requiring proteins. INF has been associated with modulated zinc transporter [17,18,19,20] and MT [21,22] expression in a variety of cell types. Inflammatory cytokines have been reported both to up- and down-regulate the expression of specific ZnT and Zip transcripts [19,23]; the net effect of the altered zinc transporter expression profile has been hypothesized to maintain or increase intracellular zinc in response to an increased demand for zinc in inflammatory conditions [19].

## 3. Zinc Status and Cytokines

The acute phase response to stress, trauma, and infection includes a transient and rapid decline in the plasma zinc concentration as a result of the redistribution of zinc into the cellular compartment. The increase in intracellular zinc is proposed to supply additional zinc for protein synthesis, neutralization of reactive nitrogen and oxygen species, and prevention of microbial invasion [24]. Zinc redistribution in inflammatory conditions appears to be mediated at least partially by cytokines; the exogenous administration of lipopolysaccharide (LPS) in healthy humans resulted in a rapid decrease in the serum zinc concentration that was preceded by prominent increases in TNF-α and IL-6 levels [25]. Cross-sectional studies support a relationship between cytokine and plasma zinc concentrations in trauma and infection. Patients with severe closed head injury exhibit hypozincemia along with a prominent cytokine and acute phase response [26]. In critically ill infected and noninfected adults assessed early after intensive care unit admission, lower plasma zinc concentrations were associated with higher illness scores and increased cytokine production [17].

### 3.1. Cytokines in Chronic Inflammation

Chronic INF is characterised by elevated production of inflammatory cytokines [27]. A relationship between zinc status and cytokine production is reported in conditions associated with chronic INF. In overweight and obese adults, participants with low dietary zinc intakes (5.7 mg/day) were found to have lower plasma zinc concentration, intracellular zinc content, and intracellular free zinc levels and upregulated IL-1α, IL-1β, and IL-6 genes compared to overweight and obese participants with zinc intakes (12.2 mg/day) that met recommended dietary requirements [28]. 

### 3.2. Human Zinc Deficiency

The generation of a variety of cytokines, including IL-1β, IL-2, IL-6, and TNF-α, is reportedly influenced by mild to moderate zinc deficiency in humans. IL-1β production was found to be higher in LPS-stimulated peripheral blood mononuclear cells (PBMC) from zinc-deficient adults (as induced by experimental diet) compared to their zinc-sufficient counterparts [29,30]. Compared to zinc-sufficient individuals (as defined by the zinc concentration of lymphocytes, granulocytes, and platelets), phytohaemagglutinin (PHA)-induced production of IL-2 was lower in PBMC of zinc deficient patients with head and neck cancer and in zinc-deficient healthy volunteers [29]. Consistent results were observed also in an elderly population, with lower IL-2 and IL-2Rα mRNA observed in PBMC isolated from zinc-deficient (defined as plasma zinc <90 µg/dL (13.8 µmol/L)) compared to zinc-sufficient subjects [31]. After a 10 week zinc-restricted (4.6 mg/day) diet, the PHA-stimulated secretion of IL-2R was reduced in PBMC of healthy men [32].

Inconsistent results have been reported for IL-6. Zinc deficiency, defined as a plasma zinc concentration <9.95 mmol/L, in Indonesian infants was accompanied by lower production of IL-6 after *ex vivo* stimulation of whole blood with LPS and PHA [33], while no significant differences between zinc-sufficient and zinc-deficient adults were observed in the production of IL-6 in PBMC stimulated with PHA alone [29]. In a similar vein, divergent results have been reported for TNF-α, which was higher in LPS-stimulated [30] and lower in PHA-stimulated [29] PBMC from zinc-deficient subjects, suggesting that the source of cell-stimulation influences cytokine production. 

### 3.3. Zinc Supplementation Studies in Humans

Human intervention studies measuring the effects of zinc on plasma cytokine concentrations or cytokine production in primary human blood cells are shown in Table 2 [34,35,36,37,38,39,40,41,42,43,44]. Supplementation with ≥45 mg zinc/day has been reported to decrease *ex vivo* generated levels of pro-inflammatory cytokine mRNA and proteins in stimulated mononuclear cells [34,35,37]. Conversely, increased cytokine concentrations have been shown in stimulated mononuclear cells isolated from populations supplemented with ≤20 mg zinc/day [39,40,41], suggesting a zinc dose-response. Measurements of plasma cytokine concentrations in response to zinc supplementation support a difference in effect depending on zinc dose; plasma concentrations of IL-6 have been shown to decrease with zinc supplementation of 45 mg/day [36] but to increase with 10 mg zinc/day [42,43]. The significance of these changes is unclear but the ability of zinc supplementation to influence cytokine concentrations in humans is consistently reported.

### 3.4. *In Vitro* Studies in Human Cells

A number of *in vitro* studies have measured inflammatory cytokine production in response to zinc depletion or supplementation in stimulated and unstimulated cells. In PHA- and phorbol myristate acetate (PMA)-stimulated HUT-78 cells, mRNA levels of IFN-γ were increased in zinc-sufficient (15 µM) compared to zinc-deficient (1 µM) cells [45]. In PHA- and PMA-activated human Jurkat T cells, supplementation with 50 or 100 µM zinc significantly reduced IFN-γ mRNA expression without affecting cell viability; in contrast, cells without stimulation did not express IFN-γ [46]. Together these results suggest that in inflammatory conditions IFN-γ gene expression is reduced both by zinc deficiency and zinc supplementation. 

After PMA or LPS stimulation, human derived-promyelocytic leukemia (HL-60) and human vascular endothelial cells cultured in 1 µM zinc (zinc-deficiency) demonstrated significantly higher generation of TNF-α and IL-1β cytokines than cells cultured in 15 µM zinc (physiologic conditions) [47]. The effect of zinc deficiency on cytokine concentrations was not apparent in unstimulated cells; before PMA or LPS stimulation, both groups of cells produced only trace amounts of TNF-α and IL-1β regardless of the zinc concentration of the media and despite adverse changes in oxidative stress markers in the zinc deficient cells [47]. Similarly, in unstimulated THP-1 monocytic cells no significant effects of the addition of 120 µM zinc on TNF-α, IL-1β, and IL-6 concentrations were observed, however zinc activated the release of IL-8 [48]. In human promonocytic HL-CZ cells, 150 µM zinc increased IL-6 as well as IL-8 levels [49], which may reflect the higher zinc concentration or cell-specific differences in cytokine release.

Cell culture studies using primary human cells demonstrate effects of zinc on cytokines that differ according to the zinc concentration and activation state of the cell (Table 3) [50,51,52,53,54,55,56]. Zinc treatment enhanced IFN-γ & IL-10 concentrations in PHA-stimulated PBMC [50] and IL-1β & TNF-α in LPS-stimulated cells, while zinc down-regulated levels of IL-1β & TNF-α in PBMC stimulated with superantigens [55]. In PBMC isolated from healthy adults and treated for 24 hours with 3 or 30 µM zinc, no difference was observed in the concentration of TNF-α, IL-1β, IL-6, IL-12 and IFN-γ; however, an increase in all cytokines was seen with ≥100 µM zinc [52], which may indicate that high extracellular zinc concentrations act as inflammatory triggers in primary mononuclear cells. 

**Table 2:** Effects of zinc supplementation in humans on plasma cytokine concentrations or cytokine release in isolated blood cells.

Author, Year	Description of Participants	Study Design/Cell Culture Conditions	Zn Dose (mg/day)	Outcome
Bao *et al.*, 2008 [34]	36 adults with sickle cell disease; M & F; 18–47 years	RCT, parallel, 13 weeks; isolated PBMCs stimulated with LPS or PHA-P for 24 h	75	Decrease in LPS-induced TNF-α & IL-1β mRNA & protein concentrations & increase in PHA-P induced IL-2 mRNA concentrations in Zn compared to placebo group
Prasad *et al.*, 2007 [35]	50 healthy older adults; M & F; 55–87 years/whole blood stimulated for 4 h & isolated PBMCs for 24 h with LPS	RCT, parallel, 52 weeks; isolated whole blood cells or PBMCs stimulated with LPS for 4 or 24 h	45	No change in % of whole blood cells positive for IL-1β or TNF-α in Zn supplement group; decrease in *ex vivo* generation of TNF-α in PBMCs from Zn supplement group
Bao *et al.*, 2010 [36]	40 healthy older adults; M & F; 56–83 years	RCT, parallel, 26 weeks	45	Decrease in plasma IL-6 concentrations in Zn supplement group
Prasad *et al.*, 2004 [37]	20 healthy adults; M & F; 19–50 years	RCT, parallel, 8 weeks; isolated PBMCs stimulated with LPS for 24 h	45	Zn reduced concentrations of LPS-induced TNF-α & IL-1β mRNAs
Raqib *et al.*, 2004 [38]	56 *Shigella*-infected children; M & F; 1–5 years	RCT, parallel, 2 weeks; isolated PBMCs stimulated with PHA for 72 h	20	No significant effects of Zn on PHA-induced release of IL-1β, IL-2, or IFN-γ
Sandstead *et al.*, 2008 [39]	54 children; M & F; 6–7 years	RCT, parallel, 10 weeks; isolated PBMC stimulated with PHA-P for 48 h	20 ^a^	Greater release of IL-2 & IFN-γ in stimulated cells derived from Zn supplemented compared to control subjects
Aydemir *et al.*, 2006 [40]	adults; M; 19–31 years	controlled before & after trial, 10 days; isolated cells stimulated with LPS for 2 h or by antigen presentation for 2 days	15	Greater release of TNF-α, IL-1β, IFN-γ in stimulated cells (monocytes, granulocytes, & T lymphocytes, respectively) derived from Zn supplemented subjects compared to placebo
Kahmann *et al.*, 2008 [41]	19 healthy older adults; M & F; 65–82 years	uncontrolled before & after trial, 48 days; isolated PBMCs stimulated with LPS or SPEA for 72 h	10	Zn supplementation resulted in lower basal IL-6, higher LPS-induced IL-6, & higher SPEA-induced TNF-α & IFN-γ concentrations
Mariani *et al.*, 2008 [42]	39 healthy older adults; M & F; 60–83 years	uncontrolled before & after trial, 48 days	10	Increase in plasma IL-6 concentrations with Zn
Mocchegiani *et al.*, 2008 [43]	110 healthy older adults; M & F; 65–85 years	uncontrolled before & after trial, 48 days	10	No change in TNF-α but increase in plasma IL-6 concentrations with Zn
Kara *et al.*, 2011 [44]	40 athletes and sedentary young adults; M; 15–17 years	controlled before & after trial, 8 weeks	5 ^b^	Zn supplementation resulted in higher serum IL-2, TNF-α & IFN-γ concentrations compared to non-supplemented individuals, irrespective of exercise

^a^ Zinc supplement provided for 5 days per week; ^b^ Supplementation dose determined as mg/kg.

**Table 3:** *In vitro* studies that report on the effects of zinc treatment on cytokine production in primary human blood cells.

Author, Year	Treatment	Outcome
Metz *et al.*, 2007 [50]	PBMC were supplemented with 30, 60 µM Zn or 1 µM TPEN for 7 days before stimulation with PHA for 24 h	PHA-stimulated IFN-γ & IL-10 concentrations were higher in cells pre-treated with 60 µM Zn
Poleganov *et al.*, 2007 [51]	PBMC were supplemented with 7.5, 15, 30 µM Zn & stimulated with IL-1β, IL-12, or IL-18 for 39 h	Zn amplified the induction of IFN-γ by IL-1β, IL-12 & IL-18
Chang *et al.*, 2006 [52]	PBMC were treated with 3, 30, 100, 300, 1000 µM Zn for 24 h	TNF-α, IL-1β, IL-6, IL-12, and IFN-γ concentrations increased at zinc concentrations ≥100 µM in combination with decreased cell proliferation
von Bulow *et al.*, 2005 [53]	PBMC were treated with 25 µM Zn plus 50 µM pyrithione & stimulated with LPS for 24 h; primary monocytes were incubated with 25, 125 µM Zn for 1 h before addition of LPS (250 ng/mL) for 24 h, or stimulated with 1 µM Zn plus pyrithione (50 µM) & LPS for 24 h	Application of zinc plus pyrithione blocked LPS-induced release of IL-1β & TNF-α in PBMC; in monocytes, 125 µM Zn significantly inhibited TNF-α release compared to controls; the application of zinc plus the pyrithione ionophore abrogated LPS-induced release of IL-1β & TNF-α
Wellinghausen *et al.*, 1997 [54]	PBMC in serum-free medium were stimulated with 100 µM Zn for 48 h	Zn increased IL-1β concentrations; Zn-induced secretion of IFN-γ was not measurable
Driessen *et al.*, 1995 [55]	PBMC were supplemented with 12.5, 25, 50, 100 µM Zn & stimulated with LPS, SEA, or SEE for 24 h (TNF-α), 48 h (IL-1β), & 72 h (IFN-γ)	IL-1β & TNF-α concentrations in LPS-stimulated cells were enhanced by the addition of Zn in a concentration-dependent manner; Zn down-regulated levels of IL-1β & TNF-α in cells stimulated with SEA & SEE superantigens
Scuderi, 1990 [ 56]	PBMC were incubated with 30, 60, 120, 250, 500, 1000, 2000 µM Zn for 18 h; in addition, cells were incubated with 63, 125, 250, 500, 1000 µM Zn plus a substimulatory concentration of LPS (0.01 pg/mL)	Addition of Zn resulted in a concentration-dependent stimulation of TNF (with a peak at 250 µM) & IL-1β (peak at 120 µM), IL-6 was unaffected by Zn; Zn & LPS in combination resulted in a synergistic stimulation of TNF but not IL-1β secretion

## 4. Immune Cells

Zinc may affect the generation of cytokines by influencing the normal development and function of immune cells. Cytokines are produced by a variety of cells, although most commonly from T lymphocytes and macrophages [11]. Experimental zinc deficiency decreases the activity of serum thymulin, which is required for the maturation of T-helper cells [57], leads to an imbalance of T-helper 1 (Th_1_) and T-helper 2 (Th_2_) functions, decreases the recruitment of T-naive cells [58], and reduces natural-killer (NK) cell lytic activity [59].

A number of studies suggest that zinc may influence the function of polymorphonuclear neutrophils (PMNs). PMNs are an important component of the acute inflammatory response, providing the primary cellular defense against bacteria in humans [60]. Beyond their traditional role as professional phagocytes, neutrophils can be induced to express a variety of cytokines, including TNF-α, IL-1β, and IL-12, and chemokines, such as IL-8 [61]. *In vitro* zinc deficiency studies have found that zinc depletion disrupts cell membrane barrier integrity and induces increases in the secretion of IL-8 and neutrophil transmigration [62]. Zinc also may modulate the oxidative burst that is generated by PMNs as part of their microbicidal activity. Stimulated PMNs released lower levels of superoxide anion (O_2_^−^) in the presence of 20 μmol extracellular zinc compared to untreated cells; in contrast, PMNs exposed to 200 μmol zinc generated higher O_2_^−^ levels [63]. Substantial evidence implicates a pathogenic role for PMN-derived oxygen metabolites in a range of disorders that are associated with perturbed zinc homeostasis and oxidative stress, including myocardial injury and arrhythmias during ischaemia and reperfusion [15,64].

Microarray analysis in the HUT-78 human T-lymphoma cell line provides evidence for a shift in global gene expression during zinc deficiency. Zinc deficient conditions affected gene expression of proteins that are associated with T-cell receptor subunits, antigen recognition molecules, adhesion molecules, and genes associated with lymphoid function. When zinc deficient HUT-78 cells were stimulated, gene expression of molecules associated with IL-1β responsiveness was increased and expression of IL-2R, IL-6R, and IL-4 was decreased compared to zinc adequate controls. An increase was observed also in the gene expression of molecules found in atherosclerosis, e.g., PTX3, which is rapidly induced by IL-1β [30].

## 5. Key Signaling Mechanisms: NF-κB and Nitric Oxide

The ability of zinc to regulate both negatively and positively the signalling pathways of TLR and the IL-1 and TNF receptors (IL-1R and TNF-R, respectively) may reflect its ability to induce or inhibit the activation of NF-κB, a ubiquitously expressed nuclear transcription factor that is critically involved in proliferation, immunity, INF, and apoptosis [15]. The TLR, IL-1R, and TNF-R signalling pathways converge on a common IκB kinase complex that phosphorylates the NF-κB inhibitory protein, IκBα, resulting in the release of NF-κB and its translocation to the nucleus [65]. 

Zinc has been depicted as both a negative and positive regulator of NF-κB. *In vitro*, incubation of HUT-78 (Th0) cells in media containing either low (1 μM) or high (50 μM or 100 μM) zinc concentrations decreased the activation of NF-κB and the expression levels of IL-2, IL-2R, and TNF-α compared to cells grown in 15 μM zinc medium. Cell growth (but not cell viability) was observed to be lower in the 1 μM, 50 μM and 100 μM zinc media, suggesting an altered cellular metabolism in cells exposed to non-physiological concentrations of extracellular zinc [66]. 

In contrast, the lipopolysaccaride (LPS)-induced activation of NF-κB was decreased in a concentration-dependent manner in human monocytes cultured in 10 μM, 20 μM, and 45 μM zinc/pyrithione (50 μM) [67]. In line with this result, HL-60 and human vascular endothelial cells cultured in 15 μM zinc demonstrated significantly increased concentrations of A20 protein after stimulation with PMA or LPS compared to stimulated cells cultured in 1 μM zinc [47]; A20 has been shown to inhibit TNF-α and IL-1β induced activation of NF-κB in endothelial cells [68], suggesting that it acts as a general inhibitor of NF-κB activation. Conversely, exposure of cultured human airway epithelial cells to 50 μM of zinc increased NF-κB-dependent transcriptional activity compared to control cells [69], indicating that the effects of zinc on NF-κB activation may be cell specific. Differences in cell type and study model, the zinc concentrations used, and the impact of various agents (such as chelating agents and zinc ionophores) on intracellular free zinc fluctuations make apparently contradictory *in vitro* observations of the effect of zinc on NF-κB activation difficult to reconcile.

*In vivo*, the effects of zinc on NF-κB activity appear to depend on the health and/or zinc status of the host. Diet-induced zinc deficiency in a murine model of polymicrobial sepsis enhanced NF-κB p65 DNA binding activity in vital organs and the expression of a range of NF-κB targeted genes known to increase systemic INF. Short term zinc repletion before the onset of sepsis significantly reduced these effects [70]. In humans, NF-κB activation and the mRNA levels of the NF-κB-regulated IL-2 cytokine and IL-2Rα receptor were found to be decreased in the peripheral blood mononuclear cells of elderly subjects with plasma zinc concentrations below the normal range (110 ± 10 µg/dL (16.8 ± 1.5 µmol/L)) compared to those with normal plasma zinc values. These effects were corrected with zinc supplementation of 45 mg/day Zn gluconate [31]. 

NO is an endogenous signalling molecule that is also involved in the inflammatory response of IL-1 and TLR ligands [71]. It is synthesized from L-arginine and O_2_ by members of the nitric oxide synthase (NOS) family of dimeric enzymes. The release of NO by the endothelium plays a key role in vascular homeostasis. Its anti-inflammatory actions include the inhibition of caspases, and thus IL-1β and IL-18 generation, and suppression of the clonal expansion of T-cells [72]. The zinc-dependent enzymes CuZnSOD and EC-SOD function to protect the cellular availability of NO, and therefore its anti-inflammatory functions, by controlling O_2_^•−^ concentrations. Excess O_2_^•−^ reacts with NO to form ONOO^−^. The ONOO^−^ anion in turn oxidizes the zinc-thiolate cluster at the dimer interface of endothelial NOS (eNOS), leading to the release of zinc and consequent dimer disruption and uncoupling of the enzyme; uncoupled eNOS demonstrates increased O_2_^•−^ production and decreased NO synthesis [73]. Numerous studies have reported that eNOS uncoupling is an important mechanism of pathologic O_2_^•−^ production in the vascular endothelium [74]. NADPH oxidase also has been proposed to play a central role in eNOS uncoupling [75]. An increased expression of the p22^phox^ subunit of NADPH oxidase has been demonstrated in the walls of human coronary atherosclerotic arteries [76], and NADPH oxidase appears able to be activated by zinc [77]. Localized zinc deficiency or the potential for zinc to be aberrantly redistributed among target proteins and intracellular compartments during the atherosclerotic process likely ameliorates this protective effect.

## 6. Cardiometabolic Disease

An altered distribution of zinc among its principal plasma proteins has been observed in atherosclerosis [15,78] and the ease with which labile zinc is transported into endothelial cells [79] suggests that the vascular endothelium may be particularly affected by perturbations in zinc homeostasis and metabolism [80]. There are conflicting reports of the relationship between atherosclerosis and zinc status, as assessed by dietary intake of zinc and/or the measurement of zinc concentrations in healthy and diseased tissues; the balance of epidemiological studies points to an association between zinc deficiency and atherosclerosis however the studies are hampered by the lack of a decisive biomarker of zinc status. Zinc has also been linked to several cardiovascular risk factors including plasma lipoprotein concentrations, haemostasis and antioxidant status [81]. Clinical trials are mainly of zinc supplementation, and these show a decrease in plasma high-density lipoprotein cholesterol concentrations leading to an increased risk of heart disease [82]. Impaired zinc homeostasis has been associated with increased levels of oxidative stress and the induction of widespread genomic and proteomic changes that relate to cardiovascular disease. 

Zinc deficiency has been suggested to exacerbate the detrimental effects of specific fatty acids, such as linoleic acid, and inflammatory cytokines, such as TNF-α, on vascular endothelial functions. Endothelial cells rendered zinc deficient demonstrated considerably higher levels of apoptotic cell death and caspase-3 activity than control cells when stimulated with linoleic acid and TNF-α. This effect was blocked by concurrent administration of zinc to the culture medium [83]. Conversely, increases in intracellular free zinc concentrations resulted in a rise in oxidative stress-related apoptosis of endothelial cells [84], highlighting that a change in the cellular zinc concentration in either direction could promote cell death in the endothelium.

Potential mechanisms of the influence of zinc on atherogenesis studied in rodent models and in cell culture include its interaction with a wide range of cellular redox and inflammatory processes, such as NF-κB, NO, PPAR, and PKC signalling pathways. Cellular zinc deficiency has been shown to upregulate NF-κB activity in endothelial cells [85] and high concentrations of NF-κB have been found to be present in the smooth muscle cells of the atherosclerotic lesion [86]. NF-κB is a component of the adhesion molecule upregulation process; is involved in the promotion of smooth muscle cell proliferation [87]; and mediates signal transduction by TLR, which play an important role in the initiation of the innate immune response and are implicated in atherogenesis [88]. The release of NO by the endothelium plays a role in vascular homeostasis. One way in which the relationship between NO and zinc may promote atherogenesis relates to Nrf2 expression in vascular cells, which is a key factor in the cellular protection against oxidative stress and INF. A release of intracellular zinc from proteins containing zinc-sulfur complexes, stimulated by inducible NOS-derived NO, has been shown to be a critical component of an Nrf2-dependent signaling pathway that activates the glutathione redox cycle in endothelial cells, ultimately protecting against oxidative damage [89].

The anti-inflammatory and antioxidant potential of zinc is supported by a recent zinc supplementation study. In a trial in healthy elderly subjects, supplementation with 45 mg Zn/day for 6 months compared to placebo was associated with an increase in plasma antioxidant power (represented by ascorbate equivalent units, U/mL) and a decrease in plasma concentrations of CRP, IL-6, macrophage chemo-attractant protein 1 (MCP-1), vascular endothelial cell adhesion molecule 1 (VCAM-1), and oxidative stress markers [36]. Taken together with the potential high-density-lipoprotein-raising effect of zinc [82], these data indicate that zinc may exert an atheroprotective effect under some conditions [36].

**Figure 1:**
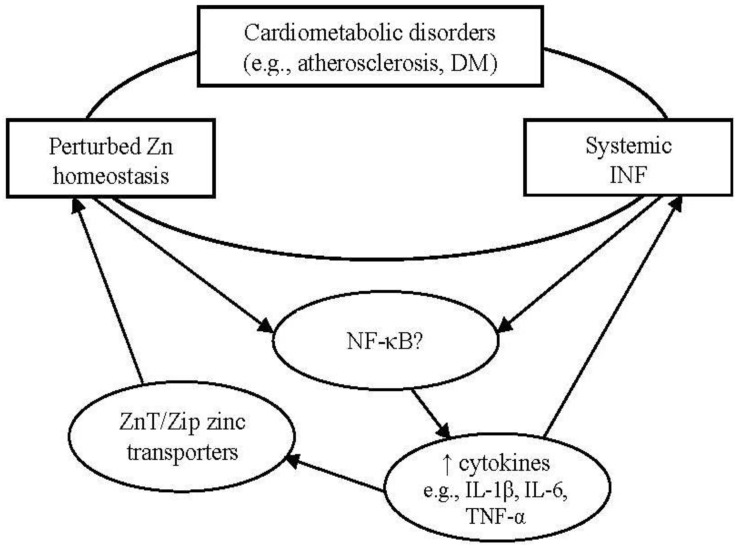
Potential interrelationship between cardiometabolic disorders, perturbed zinc homeostasis, and systemic inflammation. Cardiometabolic disorders, such as atherosclerosis and DM, often are associated with impaired zinc homeostasis and low-grade systemic INF. Depending on the health and/or zinc status of the host, zinc may enhance the expression of a range of NF-κB targeted genes known to increase systemic INF, including inflammatory cytokines. Cytokines have been shown to modulate the expression of zinc transporters, suggesting that non-resolving INF may contribute to perturbed zinc homeostasis.

Perturbed zinc homeostasis has been observed also in DM. Both Type 1 and Type 2 DM exhibit an impaired immune function as part of their pathogenesis that ultimately results in a decreased functional β-cell mass; while Type 1 DM is primarily an autoimmune disorder that leads to rapid β-cell destruction, the failure of β-cells in Type 2 DM occurs over a prolonged period and involves the chronic activation of the innate immune system [90]. The sustained or aberrant expression in DM of a number of immune mediators, including NF-κB, IL-1β and IL-6, suggests a potential interaction between the impaired immunity and the perturbed cellular zinc homeostasis associated with the disease. An overview of the proposed interrelationship between impaired zinc homeostasis, systemic INF, and cardiometabolic disorders is depicted in Figure 1.

Zinc has the ability to regulate both negatively and positively the signalling pathways of TLR and the IL-1 receptor, which may reflect its ability to induce or inhibit NF-κB activation. Increased concentrations of IL-1β have been observed in the pancreatic islet in humans with Type 2 DM [91], and human islets have been shown to respond to metabolic stress *in vitro* by increasing IL-6 release [92]. A prospective examination of the effects of IL-1β, IL-6, and TNF-α on the development of Type 2 DM found that participants with detectable levels of IL-1β and elevated concentrations of IL-6 in plasma had a threefold increased risk of developing DM compared to the reference group [93]. Both IL-1β and IL-6 are pleiotropic and are known to exert both beneficial and detrimental effects on a variety of cell types, including the pancreatic β-cells, depending on the cytokine concentration and the duration of exposure. Higher doses and longer exposure times impair glucose-stimulated insulin secretion and, at least in the case of IL-1β, increase β-cell apoptosis [94]. 

The characterizing feature of DM is the presence of chronic hyperglycemia, which is known to enhance oxidant production and impair antioxidant defense mechanisms [95,96]. Zinc supplementation has been reported to decrease cytokine levels [36] and glycemic marker concentrations [97,98], suggesting that hyperglycemia and chronic INF in type 2 DM are connected. In a preliminary evaluation of clinical trials investigating the effect of zinc supplementation on fasting blood glucose and serum insulin concentrations, a small but statistically significant reduction in fasting glucose concentrations was observed after zinc supplementation and in secondary analyses of participants with chronic metabolic disease, zinc supplementation produced a greater reduction in plasma glucose concentrations compared to the effect that was observed in healthy participants [99]. The significant albeit modest reduction in plasma glucose concentrations, suggest that zinc contributes to the management of hyperglycemia, and thereby reduces INF, in individuals with chronic metabolic disease.

β-cell destruction can be mediated by autoreactive T-lymphocytes such as CD4^+^ and CD8^+^ cells; cytokines have been shown to induce the expression of the Fas (CD95, APO-1) receptor in the β-cell, thereby sensitizing it to T-lymphocyte mediated destruction [90]. The involvement of T-cells in β-cell failure, and INF more broadly, may further implicate perturbations in zinc homeostasis in the pathogenesis of DM. Zinc deficiency is well-known to disrupt the development and function of T-cells by causing, for instance, a reduction in thymic involution [100] and a decrease in the CD4^+^ to CD8^+^ cell ratio [8], both effects that have been shown to be corrected by zinc supplementation in humans.

## 7. Conclusions

Recognition of the public health importance of zinc continues to expand, as does knowledge of the multitude of biological pathways affected by zinc and its interaction with INF. Impaired zinc homeostasis, chronic INF, and increased levels of oxidative stress feature prominently in a number of cardiometabolic diseases, including atherosclerosis and DM. Positive indications for zinc supplementation in ameliorating INF and oxidative stress in cardiometabolic disorders raise the possibility of specific therapeutic manipulation by zinc-based treatments [15,101], however caution is required in the administration of zinc in the clinical setting. Given the potential for high zinc doses to produce adverse effects, including the inhibition of T-cell functions and aberrant expression of cytokines [102] and a decrease in Cu-Zn superoxide dismutase activity [103], further well-designed randomized controlled trials are necessary to provide cogent insight into safe and desirable levels of zinc supplementation in varied populations. Additional investigations of the molecular mechanisms that underpin the sensing and distribution of zinc also are necessary to explain their effects in humans.
